# Travelling for Health

**Published:** 1857-03

**Authors:** 


### TRAVELLING FOR HEALTH.

"DEATH OF REV. LEWIS WELD.—We regret to learn, through the Hartford *Courant*, of the death of Rev. Lewis Weld, Principal of the American Asylum for the Deaf and Dumb at Hartfort, Conn. Mr. Weld was an earnest and efficient worker in the cause of Deaf-Mute education, and the members of the venerable Institution lately under his care sustain a heavy loss in his decease. He had but just returned from a European trip, undertaken for the benefit of his health, which had recently become much impaired. The voyage did not alleviate his complaint, a congestion of the lungs. For the past twenty years Mr. Weld has acted as the Principal of the Asylum."

The above is given as another of the numerous illustrations of the utter inefficiency of going abroad for diseases of the lungs. The atmosphere of steamers and sail-vessels, loaded with impurities of bilge-water, of hot steam, of cookery, their damp decks, confined promenades, shelf-bedsteads, are as well calculated to benefit invalids as the sumptuous hotels, diligence accommodations, postal and passport comforts of the continent, and yet for these, invalids leave a loved and loving home in multitudes every year, sometimes only to get home again and die, as in this case ; at others, to die in sight of home ; and often, very often, never to return.

Some of the experiences of foreign travel are detailed by an ex-editor while abroad for his health :

"It may be strange, but it is nevertheless true, that I have been as really and truly home-sick for the last three months as ever was any little girl in her first quarter at the boarding-school. If you knew how much pleasanter a life of real work and study in the United States is than this nonsensical travel and idleness, you would not be so discontented. One will only learn by experience, however ; and the best thing I expect to get, personally, out of this mission, is just this—that I will be satisfied when I get back, and never again be haunted by those intolerable longings for Europe, which tormented me in the years gone by.

"The pleasure of actually seeing celebrated places is small. It is all anticipation and memory. The real comforts of Europe don't compare with those of the United States. Everything costs just double what it does at home. The people are nowhere as good as ours. The women are uglier—the men have fewer ideas. I intended to write a book about it all ; and I thought, when I left the United States, that I would have to stretch the blanket a good deal to make out our superiority. But there is no need. The meanness, the filthy life, the stupidities of all the countries I have seen, surpassed all I expected, and all I hoped.

"Here, in Turin, which is the most beautiful city I have ever seen, I am busy learning to speak French, and studying what is popularly, but most falsely, termed the 'great world' and 'polite society.' I have dined with dukes, jabbered bad grammar to countesses, and am spunged on for seats in my opera box by counts, who smell of garlic, as does the whole country. I receive visits from other diplomates, with titles as long as a flagstaff, and heads as empty as their hearts, and find the whole concern more trashy than I had ever imagined. I must, however, keep up their miserable acquaintance, for that is the way to see the 'elephant' of European life. So I dance the dance of fools like the best of them, and return their visits sedulously, carrying about great cards, like that I inclose for your inspection.

"The pictures, the operas and ballets of Europe are good things ; the people, the governments, and the society, more contemptible than can be conceived.

"I find the idea current among the European physicians, which I have often broached to you, that chemistry is not competent to extract all the essential components of natural productions."

